# Bewegung eines Einzelkorns unter dem Einfluss kohärenter Strukturen

**DOI:** 10.1007/s00506-023-00961-1

**Published:** 2023-06-07

**Authors:** C. Sindelar, J. Schobesberger, T. Gold, K. Reiterer, D. Worf, C. Hauer, H. Habersack

**Affiliations:** 1 Department für Wasser – Atmosphäre – Umwelt, Institut für Wasserbau, Hydraulik und Fließgewässerforschung, Universität für Bodenkultur Wien, Am Brigittenauer Sporn 3, 1200 Wien, Österreich; 2 VERBUND Hydro Power GmbH, Wien, Österreich

**Keywords:** Kohärente Strukturen, Bewegungsbeginn, Zeitaufgelöstes 3D PTV, Coherent structures, Incipient motion, Time resolved 3D PTV

## Abstract

Der Einfluss von kohärenten Strukturen auf den Bewegungsbeginn eines fluvialen Einzelkorns wurde in einer grundlegenden Studie experimentell untersucht. Zur vollständigen Charakterisierung kohärenter Strukturen muss das dreidimensionale Geschwindigkeitsfeld zeitlich hochaufgelöst bekannt sein. Unter Einsatz eines tr-3D PTV-Systems (tr = zeitaufgelöst, PTV = Particle Tracking Velocimetry) konnte dies erreicht werden. Der Einfluss von Hairpin-Wirbeln und gegenläufig rotierenden Längswirbeln (VLSM) auf den Sedimenttransport wurde in mehreren Studien postuliert, mangels verfügbarer 3D-Information fehlte aber bisher ein Nachweis. In den vorliegenden „Rolling-Stones-Versuchsserien“ wurde der Bewegungsbeginn eines Einzelkorns auf glatten sowie auf rauen Sohlen untersucht. Es konnte erstmals gezeigt werden, dass sowohl Hairpin-Wirbel als auch VLSM den Bewegungsbeginn auslösen. Hairpin-Wirbel konnten zudem entgegen der gängigen Meinung auch auf rauen Sohlen nachgewiesen werden und lösten den Bewegungsbeginn aus. Die langfristig angelegte Studie soll in den nächsten Jahren auch praktische Anwendungen finden und die Genauigkeit von Sedimenttransportberechnungen in Flüssen erhöhen.

## Abstract (en)

In a fundamental study the influence of coherent structures on the incipient motion of single fluvial grain was experimentally investigated. To fully characterize coherent structures, the three-dimensional velocity field must be known with high temporal resolution. Using a tr-3D PTV system (tr = time-resolved, PTV = particle tracking velocimetry) this could be achieved. The influence of hairpin vortices and counter-rotating longitudinal vortices (VLSM) on sediment transport has been postulated in several studies, but due to the missing 3D information, evidence has been lacking. In the present “Rolling Stones test series”, the incipient motion of a single grain was investigated for smooth and rough beds. It could be shown for the first time that both hairpin vortices and VLSM trigger particle entrainment. Hairpin vortices could also be detected on rough beds, contrary to common hypotheses, and triggered particle movement. The long-term study should also find practical applications in the coming years and increase the accuracy of sediment transport calculations in rivers.

## Einleitung

Eine große Herausforderung in der Fließgewässerforschung und -praxis ist die Prognose von morphologischen Entwicklungen in Flüssen. Selbst an Flussstrecken mit hartverbauten, unbeweglichen Ufern ist das Transport‑, Mobilisierungs- und Ablagerungsverhalten von Sedimenten schwierig vorherzusagen. Die Komplexität nimmt zu, wenn Uferbauten entfernt und eigendynamische Entwicklungen mit Böschungsanrissen und Flusslaufverlagerungen initiiert werden sollen.

Der Grund für die schlechte Prognostizierbarkeit liegt zum einen an einer nicht vollständigen Datengrundlage, etwa was die Korngrößenverteilung der Sedimente von Deckschicht und Unterschicht der Sohle sowie jener des transportierten Materials betrifft. Auch mögen exakte Wasserspiegellagen und Abflüsse nicht in ausreichendem Maß bekannt sein. Doch selbst wenn diese Randbedingungen bekannt sind, ist die Wahrscheinlichkeit vorhanden, dass die Prognose fehlerbehaftet ist, und das mit Fehlern bis zu mehreren Größenordnungen (Sindelar 2022).

Die mangelhafte Vorhersagegüte liegt zum anderen vor allem auch an den zugrunde liegenden semi-empirischen Formeln für den Bewegungsbeginn und die Sedimenttransportraten.

**Figure 1:**
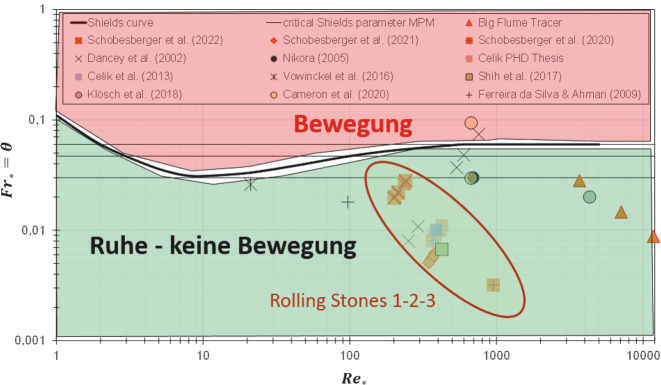

Die bahnbrechende Arbeit über den Bewegungsbeginn von Sedimenten an Flusssohlen stammt von Albert F. Shields aus dem Jahr 1936. Shields schlägt das Konzept einer kritischen Sohlschubspannung *τ*_*C*_ vor. Herrscht in einem Fluss eine Sohlschubspannung , die kleiner ist als *τ*_*C*_, ist die Sohle in Ruhe. Ist die aktuelle Sohlschubspannung τ größer als *τ*_*C*_, beginnen sich einzelne Körner zu mobilisieren. Je größer die Differenz von *τ* zu *τ*_*C*_, desto mehr Sedimente sind in Bewegung. Das berühmte Shields-Diagramm (Abb. 1) ist das Resultat zahlreicher Bewegungsbeginn-Versuche von Shields mit verschiedenen Durchflüssen, Korngrößen und Sedimentdichten (Shields 1936). Aus der Shields-Kurve kann die kritische Sohlschubspannung in dimensionsloser Form abgelesen werden. Die dimensionslose Sohlschubspannung wird mit $Fr_{*}$ bzw. *θ* bezeichnet und ist auch unter dem Namen Shields-Parameter bekannt.

Liegt die dimensionslose Sohlschubspannung für einen bestimmten Flussabschnitt bei einem Durchfluss über der Shieldskurve, ist die Sohle in Bewegung. Liegt sie darunter, ist die Sohle in Ruhe. Es findet keine Bewegung statt. Entspricht die dimensionslose Sohlschubspannung genau der kritischen Sohlschubspannung, liegt sie auf der Shields-Kurve. Es herrschen Bedingungen vor, bei denen der Sedimenttransport an der Sohle einsetzt. Der Übergang von Ruhe zu Bewegung wird bewusst als Band und nicht als Linie dargestellt, um zu verdeutlichen, dass der Bewegungsbeginn nicht von 0 auf 100 einsetzt. Zanke (2013) führte aus diesem Grund ein Bewegungsrisiko ein und weist für den Bewegungsbeginn Kurven für 1, 10 und 50 % Wahrscheinlichkeit aus.

Die einzelnen Symbole in Abb. 1, die allesamt unter der Shields-Kurve bzw. knapp darüber liegen, repräsentieren Forschungsarbeiten, die zum Thema Bewegungsbeginn gemacht wurden. Bei allen diesen Forschungsarbeiten konnte Sedimenttransport festgestellt werden, obwohl das Shields-Diagramm keine Bewegung prognostiziert. Mangels besserer Alternativen ist es aber bis heute im Einsatz und Stand des Wissens.

Welche Erklärungen gibt es dafür, dass das Shields-Diagramm teilweise unzureichende Ergebnisse liefert? Ein Grund ist, dass zur Berechnung der dimensionslosen Sohlschubspannung ein charakteristischer Korndurchmesser (meist der mittlere Korndurchmesser d_m_) verwendet wird. Es kann also durchaus sein, dass sich Steine, die größer oder gleich dem charakteristischen Korndurchmesser sind, nicht bewegen, wohl aber Steine, die kleinere Korndurchmesser haben.

Eine weitere Ursache liegt darin, dass das Shields-Diagramm gemittelte Größen von Sohlschubspannungen, Geschwindigkeiten und Wassertiefen verwendet. Aufgrund der Turbulenz fluktuieren die (sohlnahen) Geschwindigkeiten und die Sohlschubspannung und können über dem zeitlichen Mittelwert liegen. Es könnte also sein, dass zwar die gemittelte Sohlschubspannung nicht ausreicht, um die Sedimente zu bewegen, wohl aber die zu bestimmten Zeitpunkten auftretenden Sohlschubspannungsspitzen.

In den vergangenen Jahrzehnten hat sich die Forschung auf die Untersuchung sogenannter „kohärenter Strukturen“ fokussiert (Sindelar 2022). Dabei handelt es sich um zusammenhängende Wirbelstrukturen, die eine begrenzte Lebensdauer haben und ggf. auch periodisch auftreten. Ein Beispiel für kohärente Strukturen sind die Wirbel der von Kármán’schen Wirbelstraße, die sich hinter einem Zylinder in der Strömung ablösen und die je nach Reynoldszahl periodisch oder aperiodisch sind. Die räumliche Ausdehnung einer kohärenten Struktur liegt in der Regel in der Größenordnung der Geometrie, also bspw. in der Größenordnung der Wassertiefe.

Im Rahmen einer Feldstudie von Liedermann et al. (2018) wurde an der Donau östlich von Wien ein Geschiebesammler, der mit Licht und Kameras ausgestattet war, an die Sohle gesetzt. Der Durchfluss lag weit unter dem prognostizierten Durchfluss für den Bewegungsbeginn nach Shields. Die Kamerabilder belegen, dass an der Sohle immer wieder schubweise Steine bewegt werden, die kurz zur Ruhe kommen, und wenig später wieder mobilisiert werden. Das weist auf das Vorhandensein von kohärenten Strukturen hin, die bei Abflussbedingungen weit unter der Shields-Kurve eine Rolle spielen könnten.

**Figure 2:**
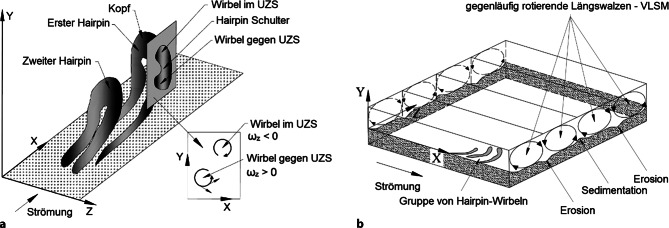

Der sogenannte Hairpin-Wirbel, der die Form einer Haarnadel hat, ist eine der ersten kohärenten Strukturen, die in Farbversuchen experimentell nachgewiesen wurden (Kline et al. 1967). Der Theorie nach entstehen Hairpin-Wirbel in der laminaren Unterschicht, wo die Geschwindigkeiten von der Sohle beginnend rasch ansteigen. Die Schichten mit höheren Geschwindigkeiten überholen die darunter befindlichen Schichten und es formt sich eine bodennahe Walze mit horizontaler Achse. Diese wird von der turbulenten Strömung nach oben gehoben und verformt sich dabei zu einem Hairpin-Wirbel. Schließlich löst sich der Hairpin auf (bursting) und der Prozess beginnt von Neuem. Hairpin-Wirbel treten häufig in Gruppen auf. In Abb. 2a sind zwei Hairpin-Wirbel dargestellt. Die zusammenhängende Struktur rotiert, sodass der Kopf des Wirbels im Uhrzeigersinn (UZS) rotiert. Schneidet man den Hairpin an seiner Schulter, rotiert der obere Teil ebenfalls im UZS, während der untere Teil gegen den Uhrzeigersinn rotiert. Eine Gruppe von Forschern konnte während des Bewegungsbeginns eines Einzelkorns zwei gegenläufige Wirbel in einem Vertikalschnitt experimentell nachweisen (Dwivedi et al. 2011; Shih et al. 2017; Wu und Shih 2012), analog zu Abb. 2a. Sie schlossen daraus, dass diese zwei Wirbel auf das Vorhandensein eines Hairpin-Wirbels hindeuten, konnten dies aber mangels 3D-Geschwindigkeitsmessungen nur mutmaßen. Gruppen von Hairpin-Wirbeln werden in der Literatur gelegentlich als Large Scale Motions (LSM) bezeichnet, um sie von noch größeren Strukturen – Very Large Scale Motions (VLSM) – abzugrenzen (Adrian und Marusic 2012). Unter VLSM versteht man gegenläufig rotierende Längswalzen (Abb. 2b). Adrian und Marusic (2012) postulierten, dass an jenen Stellen, wo die Längswalzen Richtung Boden rotieren, Erosion stattfindet, während sich Sedimente dort ablagern, wo die Längswalzen nach oben rotieren (vgl. Abb. 2b).

Im Wasserbaulabor des Instituts für Wasserbau, Hydraulik und Fließgewässerforschung der Universität für Bodenkultur Wien wurde eine grundlegende Studie durchgeführt. Die sogenannten „Rolling-Stones-Versuche“ stellen eine Versuchsserie zum Bewegungsbeginn eines Einzelkorns bei Bedingungen weit unter der Shields-Kurve dar. Unter dem Einsatz von Mess- und Auswertemethoden auf dem neuesten Stand der Technik konnte das 3‑dimensionale Strömungsfeld vor, während und nach dem Bewegungsbeginn zeitaufgelöst in einem Messvolumen gemessen und auf kohärente Strukturen untersucht werden. Das Ziel der in diesem Beitrag zusammengefassten Rolling-Stones-Versuchsserie war zum einen, den Einfluss von kohärenten Strukturen auf den Bewegungsbeginn zu untersuchen. Zum anderen sollte die gängige Hypothese überprüft werden, ob Hairpin-Wirbel bei rauen Sohlen entstehen, weil zu deren Bildung eine laminare Unterschicht notwendig ist, die bei rauen Sohlen fehlt.

## Messtechnik und Versuchs-Setup

In diesem Abschnitt wird zunächst die Messtechnik der zeitaufgelösten 3D Particle Tracking Velocimetry (time resolved 3D-PTV = tr-3D-PTV) kurz erläutert, die auch als tr-tomo-PTV bekannt ist (Elsinga et al. 2006; Wieneke 2008; Schanz et al. 2016). Es handelt sich um ein berührungsloses Verfahren, bei dem keine Sonde die zu messende Strömung stört. Eine detaillierte Beschreibung dieser Methode und der zum Einsatz kommenden Geräte findet sich in Schobesberger et al. (2020, 2021) sowie im Beitrag von Gold et al. (2023; in diesem Heft).

**Figure 3:**
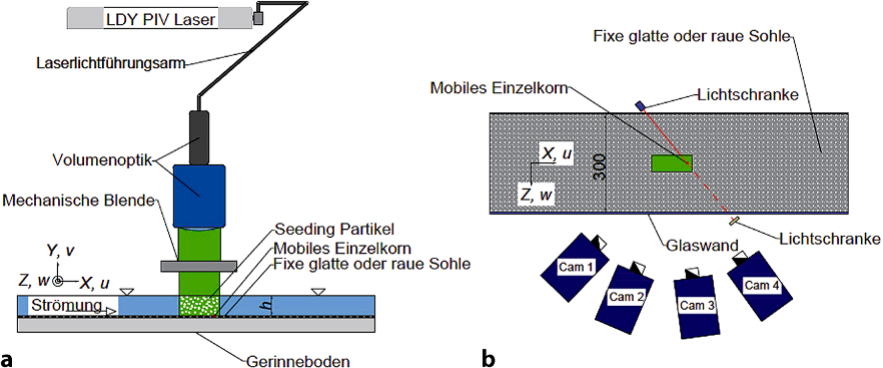

Bei den Rolling-Stones-Versuchen kam ein tr-3D-PTV-System mit vier Highspeed Kameras zum Einsatz, die auf einer Seite einer Glasrinne linear angeordnet waren (Abb. 3). Die Kameras hatten einen unterschiedlichen Blickwinkel auf das Messvolumen, in dem die zeitaufgelösten 3D-Geschwindigkeiten gemessen werden sollten. Vor den Strömungsmessungen musste das System kalibriert werden. Dazu wurde im Bereich des Messvolumens eine 3D-Kalibrierplatte eingesetzt, die zwei verschiedene Ebenen besaß. Als Ergebnis erhielt man eine Zuordnung von den Raumkoordinaten des Messvolumens zu den Pixelkoordinaten für jede Kamera.

Ein LDY-Laser erzeugte einen Laserstrahl, der über einen Laserlichtführungsarm und eine Volumenoptik in ein Volumen aufgefächert wurde. Das Laserlicht leuchtete das Messvolumen in der Strömung aus. In der Strömung befanden sich sogenannte Seeding-Partikel, die ohne starke Lichtquelle mit freiem Auge nicht sichtbar sind. Es handelte sich um Polyamid-Partikel mit einem Durchmesser von 50 Mikrometern, die sich aufgrund der geringen Größe und Dichte mit derselben Geschwindigkeit wie die Strömung bewegen. Die High-Speed-Kameras wurden mithilfe einer Synchronisationseinheit zeitgleich ausgelöst und machten mit der vorgegebenen Aufnahmerate Fotos.

Je nach Versuch betrug die Partikeldichte 0,03–0,04 ppp (particles per pixel). Jede Kamera nahm etwa 6000 Bilder mit einer Aufnahmefrequenz von 800 Hz auf. Daraus ergeben sich je nach Versuch Aufnahmedauern von rund 7,5 s. Aufnahmefrequenz und Aufnahmedauer wurden dabei durch die Größe der internen Kameraspeicher limitiert.

Für die Auswertung der Kamerabilder wurde der Algorithmus „Shake-the-Box“ (STB, Schanz et al. 2016) gewählt. STB ist früheren Particle-Tracking-Methoden hinsichtlich möglicher Partikeldichten und Genauigkeit deutlich überlegen. Höhere Partikeldichten entsprechen einer höheren räumlichen Auflösung. Das Ergebnis von STB sind zahlreiche Partikel-Trajektorien im Messvolumen, deren 3D-Geschwindigkeiten pro Zeitschritt auf ein regelmäßiges Raster interpoliert werden können. Auf diese Art und Weise wurden räumliche Auflösungen von 1,5 × 1,5 × 1,5 mm^3^ erzielt.

Abb. 3 zeigt den Versuchsaufbau im Längenschnitt (a) und im Grundriss (b). In einer Glasrinne wurde ein einzelner fluvialer Stein (Einzelkorn) auf eine fixe Sohle gelegt, die entweder glatt oder rau war. Die raue Sohle bestand aus Kies mit Korngrößen von 5,6–6 mm, die auf eine ebene Sohle geklebt waren. Es wurden Strömungsbedingungen hergestellt, die weit unter der Shieldskurve liegen. Es wurde so lange gewartet, bis der Stein mobilisiert wurde. Dieser Vorgang dauerte zwischen 30 s und mehreren Minuten. Eine Lichtschranke, die der Stein bei Bewegung auslöste, triggerte die tr-3D-PTV-Messungen.

Die Rolling-Stones-Versuchsserie bestand aus drei Settings, deren wichtigste Parameter in Tab. 1 zusammengefasst sind.

**Table 1:** 

Setting	d (mm)	Sohle	h (m)	U (m/s)	$Fr_{*}$	$Re_{*}$	Referenz
1	28	Glatt	0,171	0,44	0,003	278	Schobesberger et al. (2020)
2	12,7	Glatt	0,126	0,52	0,006	217	Schobesberger et al. (2021)
3	6	Rau	0,053–0,059	0,46–0,51	0,02–0,03	236–284	Schobesberger et al. (2022)

*d* Mittelwert der drei Steinachsen, *h* Wassertiefe, *U* Geschwindigkeit in Hauptfließrichtung

**Figure 4:**
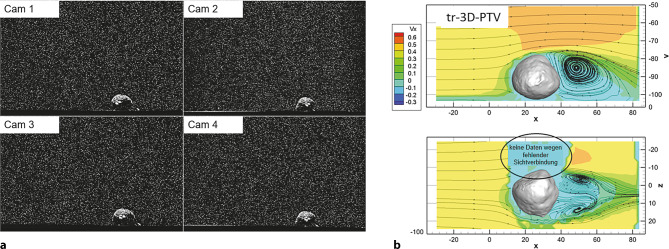

In Abb. 4a sind zur Veranschaulichung die vier Bildaufnahmen der Highspeed-Kameras zu einem Zeitpunkt dargestellt. Es handelt sich um die Versuchsserie Rolling Stones 1, bei der ein fluviales Einzelkorn auf einer glatten Sohle positioniert war. Die weißen Punkte sind die Seeding-Partikel in der Strömung, die mit freiem Auge nicht sichtbar sind, sondern nur mit einer starken Lichtquelle. Abb. 4b zeigt die zugehörige zeitlich gemittelte Geschwindigkeit U (m/s) in Hauptfließrichtung im Vertikalschnitt (oben) und im Horizontalschnitt (unten) auf halber Steinhöhe. Im Horizontalschnitt fehlen hinter dem Stein (aus Sicht der Kameras) die Geschwindigkeitsdaten. Da es sich um ein optisches Messverfahren handelt, können Geschwindigkeiten nur in jenen Bereichen ermittelt werden, zu denen die Kameras optischen Zugang haben.

## Auswertemethoden

Die „Proper Orthogonal Decomposition“ (POD) ist eine Methode zur Zerlegung eines zeitaufgelösten Strömungsfelds in einem Messvolumen in einzelne Modi (Sindelar 2022; Schobesberger et al. 2021). Aus dem 3D-Geschwindigkeitsfeld, das zu *M* Zeitpunkten („Snapshots“) vorliegt, wird die Matrix **U** gebildet und das Eigenwertproblem in Gl. 1 gelöst. Die Eigenvektoren α_k_bezeichnet man als temporäre Modi.

1 $$\boldsymbol{U}^{T}\mathbf{U}\boldsymbol{\alpha }_{\boldsymbol{k}}=\lambda _{k}\boldsymbol{\alpha }_{\boldsymbol{k}}$$

Φ_k_ aus Gl. 2 sind die zugehörigen räumlichen Modi.

2 $$\boldsymbol{\Phi }_{\boldsymbol{k}}:=\frac{\mathbf{U}\boldsymbol{\alpha }_{\boldsymbol{k}}}{\left|\left|\mathbf{U}\boldsymbol{\alpha }_{\boldsymbol{k}}\right|\right|}$$

Sortiert man die Eigenwerte *λ*_*k*_ ihrer Größe nach, enthält der erste Mode die meiste Energie, der zweite Mode die zweitmeiste usw. Das Geschwindigkeitsfeld lässt sich als Linearkombination mehrerer Modi approximieren. Je mehr Modi man zur Rekonstruktion verwendet, umso besser ist die Approximation. Allerdings wird die POD in der Regel dafür eingesetzt, das Strömungsfeld mit möglichst wenigen Modi zu rekonstruieren. Durch das Weglassen der höheren, weniger energiereichen Modi werden unkorrelierte Signale aus der Strömung gefiltert. Das erleichtert die Untersuchung großskaliger, energiereicher Strukturen.

Um im zeitaufgelösten Strömungsfeld kohärente Wirbelstrukturen erkennen zu können, wird häufig das Q‑Kriterium verwendet (Hunt et al. 1988). Dominiert in einem Bereich der Strömung die Rotation gegenüber der Scherung, ist dies ein Indiz für Wirbelstrukturen. In diesem Fall ist Q größer als 0. Je größer Q ist, desto dominanter ist die Rotation. Wirbelstrukturen können alternativ auch anhand der 3D-Wirbelstärke berechnet werden (Schobesberger et al. 2022). Wirbelstrukturen können visualisiert werden, indem man einen festen Wert für Q oder die 3D-Wirbelstärke wählt und in einer geeigneten Software (z. B. in TecPlot, Paraview etc.) Isoflächen davon darstellt.

Die Wirbel-Kriterien können für zeitaufgelöste Strömungsfelder berechnet werden. Wurde dieses Strömungsfeld zuvor mittels POD gefiltert, ist das Detektieren und Visualisieren von Wirbelstrukturen einfacher.

## Ergebnisse

Bei der Rolling-Stones-Versuchsserie 1 zum Bewegungsbeginn eines fluvialen Einzelkorns auf einer glatten Sohle konnte nachgewiesen werden, dass eine Gruppe von Hairpin-ähnlichen Wirbeln beim Bewegungsbeginn über den Stein wanderte (Schobesberger et al. 2020). Diese Versuchsserie wurde durch numerische Simulationen ergänzt (Yücesan et al. 2022; Tritthart et al. 2023; in diesem Heft). Bei dieser Versuchsserie wurde das gemessene 3D-Strömungsfeld noch nicht mittels POD gefiltert. Um die Wirbelstrukturen noch besser sichtbar machen zu können, wurde bei der Versuchsserie 2 das gemessene 3D-Strömungsfeld mittels POD gefiltert. Anhand der visualisierten Wirbel konnte nachgewiesen werden, dass das Einzelkorn bei Bewegungsbeginn genau zwischen zwei nach unten rotierenden Längswalzen lag (vgl. Abb. 2b). Die Vermutung von Adrian und Marusic (2012) über den Zusammenhang von VLSM und Erosion/Sedimentation konnte daher anhand der rekonstruierten dreidimensionalen Wirbelstruktur bestätigt werden (Schobesberger et al. 2021).

**Figure 5:**
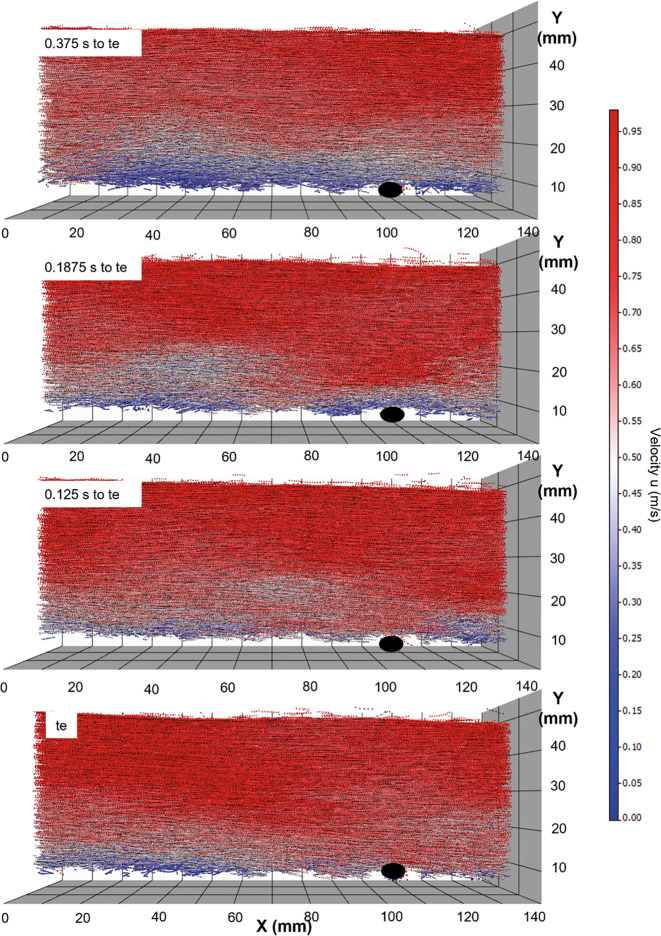

Bei der Versuchsserie 3 wurde der Bewegungsbeginn eines Einzelkorns auf einer rauen Sohle untersucht. Dabei wurde auch der Shields-Parameter $\theta =Fr_{*}$ variiert. Abb. 5 zeigt eine räumliche Darstellung der Partikeltrajektorien zu vier Zeitpunkten. Die Fließrichtung ist von links nach rechts. Die raue Sohle ist nicht sichtbar, jedoch indirekt anhand der unregelmäßigen unteren Berandung der Trajektorien erkennbar. Das Einzelkorn, das bewegt wird, ist in schwarz dargestellt. Die Farben der Trajektorien stellen die Geschwindigkeit U (m/s) in Fließrichtung dar. Weiße Bereiche repräsentieren die mittlere Fließgeschwindigkeit. Rot bzw. blau bedeutet, dass die Geschwindigkeit größer bzw. kleiner als die mittlere Geschwindigkeit ist. Der Zeitpunkt des Bewegungsbeginns ist mit te gekennzeichnet. Das oberste Bild in Abb. 5 ist 0,375 s vor dem Bewegungsbeginn, die beiden mittleren Bilder sind 0,1875 s bzw. 0,125 s vor Bewegungsbeginn und das unterste Bild stellt das Geschwindigkeitsfeld bei Bewegungsbeginn dar.

In der Bildabfolge von Abb. 5 ist klar erkennbar, dass vor dem Bewegungsbeginn die Geschwindigkeiten flussauf des Steins unter oder im Bereich der mittleren Fließgeschwindigkeit liegen. Zu Bewegungsbeginn ist der gesamte Bereich bis zur Wassertiefe tiefrot, was bedeutet, dass die Geschwindigkeiten über der mittleren Fließgeschwindigkeit liegen.

**Figure 6:**
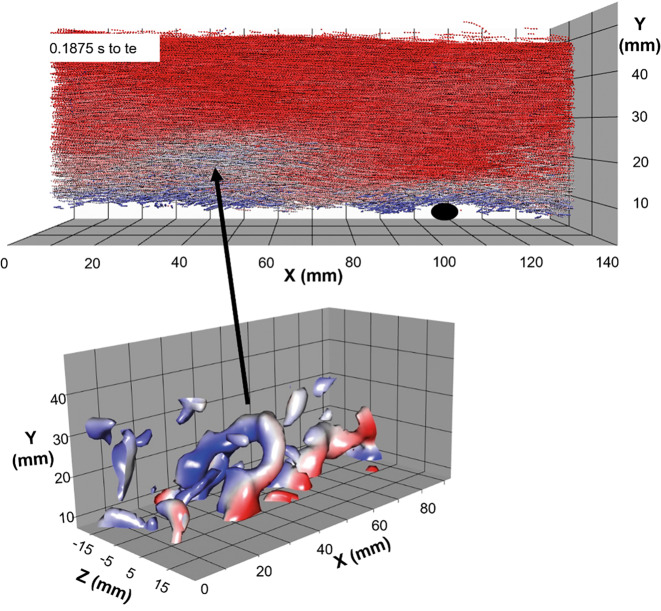

In Abb. 6 oben ist noch einmal das Strömungsfeld 0,1875 s vor Bewegungsbeginn dargestellt. Darunter sind die zu diesem Zeitpunkt existierenden Wirbelstrukturen anhand der 3D-Wirbelstärke als Isoflächen dargestellt. Ein Hairpin-Wirbel ist klar erkennbar, der in einer langsamen Strömungszone liegt.

**Figure 7:**
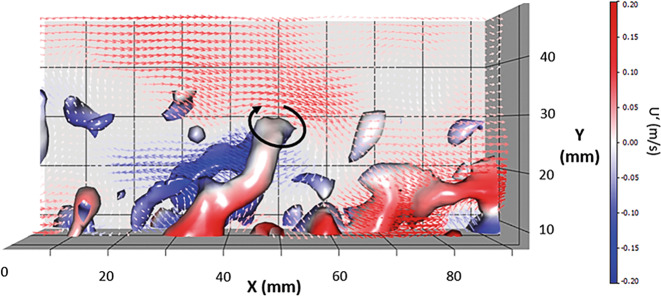

In Abb. 7 ist ersichtlich, warum das Auftreten eines Hairpin-Wirbels mit langsamen Geschwindigkeiten einhergeht. Neben den visualisierten Wirbelstrukturen sind die Geschwindigkeitsfluktuationen U′ und V′ mittels Vektoren dargestellt. U′ bzw. V′ ergeben sich aus der Differenz der Momentangeschwindigkeit U bzw. V vom zeitlichen Mittelwert. Die Farben der Vektoren repräsentieren die Geschwindigkeitsfluktuationen U′ (m/s). Am Kopf des Hairpin-Wirbels rotiert die Strömung gegen die Fließrichtung, weshalb unterhalb des Kopfs die Geschwindigkeitsfluktuationen kleiner 0 sind. Anders formuliert ist dort die momentane Strömungsgeschwindigkeit U kleiner als das zeitliche Mittel.

Die Abb. 5, 6 und 7 zeigen das Strömungsfeld bei Bewegungsbeginn eines Einzelkorns auf einer rauen Sohle für einen konkreten Versuchslauf. Insgesamt wurden 50 Versuche bei verschiedenen Shields-Parametern durchgeführt, die alle ein ähnliches Resultat lieferten. Auch über rauen Sohlen traten Hairpin-Wirbel auf, die den Bewegungsbeginn auslösten.

## Schlussfolgerungen

In diesem Beitrag wurde die Rolling-Stones-Versuchsserie vorgestellt, die eine grundlegende Studie zum Zusammenhang von kohärenten Wirbelstrukturen und dem Bewegungsbeginn eines Einzelkorns darstellt. Unter der Verwendung eines räumlich und zeitlich hochaufgelösten Messverfahrens (tr-3D-PTV) konnte in einem Messvolumen das 3D-Geschwindigkeitsfeld berührungslos gemessen werden. Daraus konnten 3D-Wirbelkriterien berechnet und anhand von Isoflächen visualisiert werden. Bei den drei Versuchsserien lag ein Einzelstein entweder auf einer glatten oder rauen Sohle. Die Fließbedingungen lagen dabei weit unter dem kritischen Shields-Parameter. Ohne zeitaufgelöste 3D-Geschwindigkeiten konnte der postulierte Zusammenhang zwischen Hairpin-Wirbeln und VLSM auf den Bewegungsbeginn bisher nicht nachgewiesen werden. In der Rolling-Stones-Versuchsserie gelang der erstmalige Nachweis, dass Hairpin-Wirbel sowohl bei glatter als auch bei rauer Sohle auftreten und den Bewegungsbeginn eines Steins verursachen. Ebenso konnte gezeigt werden, dass gegenläufig rotierende Längswalzen (VLSM) den Bewegungsbeginn auslösten. Die gängige Hypothese, dass Hairpin-Wirbel bei rauen Sohlen nicht existieren, konnte anhand der Versuchsserie 3 über eine raue Sohle widerlegt werden.

Die grundlegende Studie liefert eine Erklärung für Sedimentbewegungen an Flusssohlen weit unter kritischen Shields-Bedingungen. Das mittelfristige Ziel der Langzeitforschung ist es, die Ergebnisse der Grundlagenforschung in ein praktisch anwendbares Modell zu transferieren.
